# Management of Multidrug-Resistant Infections in ICUs: A Narrative Review of Pharmacological and Non-pharmacological Interventions

**DOI:** 10.7759/cureus.113404

**Published:** 2026-07-26

**Authors:** Chetan Chandrashekar, Mansiben Patel, Herrieth Jandwa, Amardeepsinh Jadeja, Chaitanya Kumar Javvaji

**Affiliations:** 1 Internal Medicine, Davao Medical School Foundation, Davao, PHL; 2 Internal Medicine, Spartan Health Sciences University, Vieux Fort, LCA; 3 Internal Medicine, Kilimanjaro Christian Medical University College, Kilimanjaro, TZA; 4 Internal Medicine, Smt. Nathiba Hargovandas Lakhmichand (NHL) Municipal Medical College, Ahmedabad, IND; 5 Pediatrics, Motherness Fertility, Women and Children's Hospital, Bhimavaram, IND

**Keywords:** antimicrobial resistance, antimicrobial stewardship, infection prevention and control, pharmacological management, vancomycin resistant

## Abstract

Multidrug-resistant (MDR) infections in ICUs are a growing global health challenge associated with increased morbidity, mortality, prolonged hospitalization, and rising healthcare costs. Critically ill patients are particularly vulnerable because of immune dysfunction, invasive procedures, prolonged ICU stays, and frequent exposure to broad-spectrum antibiotics, all of which increase susceptibility to MDR infections and antimicrobial selection pressure. Common MDR pathogens in ICU settings include *Acinetobacter baumannii*, *Klebsiella pneumoniae*, *Pseudomonas aeruginosa*, methicillin-resistant *Staphylococcus aureus* (MRSA), vancomycin-resistant *Enterococcus* (VRE), and carbapenem-resistant *Enterobacterales*. Major resistance mechanisms include β-lactamase and carbapenemase production, efflux pump overexpression, reduced membrane permeability, target modification, biofilm formation, and horizontal gene transfer. Effective management of MDR infections in ICUs requires an integrated, evidence-based approach combining early recognition, microbiological diagnosis, optimized antimicrobial therapy, and strict infection-prevention strategies. Pharmacological management relies on appropriate empiric therapy, antimicrobial stewardship, therapeutic drug monitoring, and pharmacokinetic/pharmacodynamic optimization to improve treatment efficacy while minimizing toxicity and resistance development. Newer antimicrobial agents, including β-lactam/β-lactamase inhibitor combinations and cefiderocol, have expanded treatment options for resistant resistant Gram-negative infections. Non-pharmacological interventions such as hand hygiene, environmental disinfection, surveillance cultures where indicated, contact precautions, and multidisciplinary infection-control programs remain essential in reducing transmission. Emerging innovations, including artificial intelligence-guided antimicrobial selection, rapid diagnostic technologies, microbiome-based therapies, and bacteriophage therapy, show promise for future management. A multidisciplinary strategy integrating prevention, stewardship, and ongoing research is essential to reduce the MDR burden in ICUs and preserve antimicrobial effectiveness.

## Introduction and background

Epidemiology and global burden of multidrug-resistant (MDR) infections in ICUs

MDR and pan-drug-resistant (PDR) bacteria are not the same as extensively drug-resistant (XDR) bacteria. MDR bacteria are resistant to one or more antimicrobial agents, whereas PDR bacteria are resistant to all antimicrobial agents [1]. This difference is primarily interpreted as the degree of drug resistance of the bacterium; consequently, XDR bacteria fall somewhere in the middle of these two groups. Definitions are sometimes based on more general categories of antimicrobials rather than individual agents, which reduces the number of bacteria that fulfill the criteria.

Antimicrobial Resistance (AMR): Financial and Global Impact

AMR reduces agricultural output, necessitates more expensive and intensive care, and lowers patient or caregiver productivity because of prolonged hospital stays, all of which have a significant financial impact on countries’ economies and health systems [2]. AMR affects countries across all income levels. Lack of access to clean water, sanitation, and hygiene (WASH) for people and animals; inadequate infection prevention and control (IPC) in homes, hospitals, and farms; lack of affordable, high-quality vaccines, diagnostics, and medications; and limited awareness, knowledge, and enforcement of relevant laws are all contributing factors [2]. People living in low-resource settings and disadvantaged groups are most affected by the causes and effects of AMR [2].

WHO Bacterial Priority Pathogens List (BPPL) 2024

In the worldwide battle against antibiotic resistance, the 2024 WHO BPPL is a crucial instrument. The list divides these pathogens into critical, high, and medium priority groups to guide research and development (R&D) and public health initiatives. The WHO BPPL emphasizes the need for regionally appropriate strategies and serves as a roadmap for prioritizing R&D and funding in AMR [3].

Common MDR Pathogens in ICU Settings

According to CDC data, ICU settings have a higher incidence of multidrug-resistant organisms (MDROs) than other hospital settings [4]. Surveillance reports identify both Gram-negative and Gram-positive bacteria as prevalent MDR pathogens in ICU settings [4]. Table 1 summarizes the WHO BPPL 2024.

**Table 1:** WHO bacterial priority pathogens list 2024. Source: WHO. Bacterial Priority Pathogens List, 2024. Geneva: WHO; 2024.

Priority Group	Pathogen	Resistance Pattern
Critical priority	Acinetobacter baumannii	Carbapenem-resistant
Critical priority	Enterobacterales	Third-generation cephalosporin-resistant
Critical priority	Enterobacterales	Carbapenem-resistant
Critical priority	Mycobacterium tuberculosis	Rifampicin-resistant
High priority	*Salmonella* Typhi	Fluoroquinolone-resistant
High priority	*Shigella* spp.	Fluoroquinolone-resistant
High priority	Enterococcus faecium	Vancomycin-resistant
High priority	Pseudomonas aeruginosa	Carbapenem-resistant
High priority	Non-typhoidal *Salmonella*	Fluoroquinolone-resistant
High priority	Neisseria gonorrhoeae	Third-generation cephalosporin-resistant and/or fluoroquinolone-resistant
High priority	Staphylococcus aureus	Methicillin-resistant
Medium priority	Group A streptococci	Macrolide-resistant
Medium priority	Streptococcus pneumoniae	Macrolide-resistant
Medium priority	Haemophilus influenzae	Ampicillin-resistant
Medium priority	Group B streptococci	Penicillin-resistant

Among Gram-negative pathogens, *Acinetobacter baumannii* is particularly prevalent in immunocompromised people and is linked to respiratory, urinary tract, and bloodstream infections (BSI) [4]. *Klebsiella pneumoniae* is linked to meningitis, BSI, wound or surgical site infections, and ventilator-associated pneumonia [4]. *Pseudomonas aeruginosa* is associated with wound and BSI and is characterized by both intrinsic and acquired resistance mechanisms [4]. Carbapenem-resistant Enterobacterales (CRE) represent a group of gut bacteria, including *Escherichia coli* and *Klebsiella pneumoniae*, that have developed resistance to carbapenems [4].

Among Gram-positive organisms, methicillin-resistant *Staphylococcus aureus* (MRSA) is commonly linked to pneumonia, BSI, sepsis, and death; the CDC encourages clinicians to consider MRSA in soft tissue infections compatible with *Staphylococcus aureus* [5]. Vancomycin-resistant Enterococcus (VRE) is common in hospital settings and is associated with urinary tract, bloodstream, and wound infections that are resistant to vancomycin [4].

Data from WHO, CDC, and European Centre for Disease Prevention and Control (ECDC) demonstrate that AMR is a global health emergency. Globally, bacterial AMR was associated with more than 4.7 million deaths in 2021, while in the United States, more than 2.8 million antimicrobial-resistant infections and more than 35,000 related deaths occur annually [2,5]. With the rise in incidence, MDR infections significantly increase patient morbidity, contributing to organ failure, the need for mechanical ventilation, and the need for extensive medical therapy [2,4].

Risk Factors and Contributing Variables

MDR infections arise from a complicated interaction between patient, healthcare, and system-level factors. At the patient level, immunosuppression, severe underlying disease, and malnutrition significantly increase susceptibility [2,5]. At the healthcare level, long hospital stays, numerous invasive procedures, and previous exposure to broad-spectrum antibiotics all increase the risk of infection. Invasive devices such as urinary catheters, central lines, and mechanical ventilators also establish pathways for infection [2,5]. At the system level, inadequate infection prevention and control measures, misuse or overuse of antibiotics, and the absence of antimicrobial stewardship (AS) programs collectively create an environment in which resistant organisms can emerge and spread unchecked [2].

## Review

Mechanisms and drivers of AMR in critical care

Enzymatic Degradation

One of the most common resistance mechanisms is the enzymatic destruction of antibiotics by bacterial enzymes. The β-lactam ring found in carbapenems, cephalosporins, and penicillins is hydrolyzed by β-lactamases, rendering these antibiotics inactive. By hydrolyzing cephalosporins and penicillins, extended-spectrum β-lactamases (ESBLs) provide resistance to broad-spectrum β-lactams. Because they render carbapenems, which are often considered last-resort antibiotics, inactive, carbapenemases such as New Delhi metallo-β-lactamase (NDM), *Klebsiella pneumoniae* carbapenemase (KPC), and OXA-48 are especially dangerous.

These enzymes can spread rapidly throughout bacterial populations, as they are commonly encoded on mobile genetic elements [6]. This enzymatic degradation has been a major driver in the emergence of carbapenem-resistant *Acinetobacter baumannii* and *Klebsiella pneumoniae* in ICUs and hospital settings worldwide [6]. In *Acinetobacter baumannii*, aminoglycoside-modifying enzymes broaden the resistance profile further, working with OXA-type carbapenemases to reduce available treatment options [6].

Efflux Pumps and Reduced Permeability

Efflux pumps are transmembrane protein complexes that actively eliminate antibiotics from bacterial cells, reducing their activity. ATP-binding cassette (ABC), major facilitator superfamily (MFS), resistance-nodulation-division (RND), small multidrug resistance (SMR), and multidrug and toxic compound extrusion (MATE) are major pump families. RND pumps, such as MexAB-OprM in *Pseudomonas aeruginosa* and AcrAB-TolC in E. coli, have been thoroughly investigated in the context of MDR [7]. In MexAB-OprM, the periplasmic adaptor protein (MexA), outer membrane channel (OprM), and inner membrane transporter (MexB) form a channel that spans the entire bacterial envelope. The proton motive force causes conformational changes that allow antibiotics and other substrates to exit the cell.

Resistance to antibiotics, including β-lactams, fluoroquinolones, and tetracyclines, can result from mutations in regulatory genes such as mexR or acrR that encourage the overexpression of efflux pump genes. Because these pumps export several substrates, MDR infections are challenging to treat [7]. Antibiotic penetration is also restricted by reduced permeability of the bacterial outer membrane as a result of porin protein loss or modification. These linked mechanisms are often seen in Gram-negative organisms, such as *Pseudomonas aeruginosa*, *Acinetobacter baumannii*, and *Klebsiella pneumoniae*, and underpin the resistance profiles commonly seen in critical care infections [8]. In difficult-to-treat *Pseudomonas aeruginosa* (DTR-PA), target-site mutations in GyrA and ParC add to fluoroquinolone resistance, working with efflux overexpression and porin loss to create multimechanistic resistance [7,8].

Target Modification

Resistance can also arise from structural changes in antibiotic targets, which reduce drug-binding affinity, thus neutralizing the antibiotic’s effect. For example, MRSA carries altered penicillin-binding proteins (PBPs), particularly PBP2a encoded by the mecA gene, which have reduced affinity for β-lactam antibiotics. This allows MRSA to survive despite β-lactam treatment [7,9]. Similar target alterations can occur in other pathogens, affecting ribosomes, DNA gyrase, or RNA polymerase, conferring resistance to macrolides, fluoroquinolones, and rifamycins. Similarly, VRE uses target modification to reduce vancomycin-binding affinity by more than 1,000-fold by replacing the D-Ala-D-Ala terminus of its peptidoglycan precursor with D-Ala-D-Lac through vanA/vanB genes [7,9].

Biofilm Formation

Biofilms are organized bacterial communities that are shielded from antimicrobial agents and host defenses by a self-produced extracellular matrix. By restricting drug penetration, slowing growth rates, and promoting horizontal gene transfer (HGT) of resistance elements, biofilm-associated cells demonstrate phenotypic tolerance to antibiotics. In ICU settings with indwelling devices such as ventilators and catheters, biofilm-associated infections are especially troublesome. Biofilms make it more difficult to eradicate pathogens [9].

Horizontal Gene Transfer

HGT is fundamental to the rapid dissemination of AMR genes among bacterial populations in ICUs. The primary mobile genetic elements that enable HGT are plasmids, transposons, and integrons. Plasmids are extrachromosomal DNA entities that frequently harbor numerous resistance genes and can be exchanged between bacteria through conjugation. Transposons are DNA sequences that can relocate inside and among genomes, occasionally transporting resistance determinants that may integrate into plasmids or chromosomes. Integrons serve as gene acquisition and expression mechanisms that incorporate gene cassettes, frequently encoding resistance traits, into bacterial genomes [10].

ICU-Specific Drivers of Resistance

In the ICU environment, several interacting factors exacerbate the development and dissemination of antibiotic resistance. First, the excessive and improper use of broad-spectrum antibiotics exerts selective pressure that promotes the proliferation of resistant microbes. Critically ill patients often undergo empiric and extended antibiotic therapy due to the urgency of infections and the complexity of their illnesses, resulting in increased selection for resistance. The indiscriminate use of antibiotics exacerbates the risk of colonization and infection by MDROs [11,12].

Second, cross-transmission via the ICU environment is a significant driver of resistance spread. ICU surfaces and equipment harbor resistant organisms, and shared healthcare areas can disseminate pathogens, particularly when biofilms form on dry surfaces and bacteria survive and resist disinfectants. Patients admitted to beds previously occupied by carriers of resistant bacteria are more likely to acquire the same strains, making rigorous cleaning practices essential [13]. Healthcare workers may act as vectors in transmitting resistant organisms through direct contact, particularly via poor hand hygiene and inadequate adherence to barrier precautions. The ICU environment, with its high patient acuity, invasive devices, and frequent antibiotic use, creates conditions conducive to resistance emergence and dissemination [14]. Additional factors influencing transmission include understaffing, heavy workloads, and inadequate training, particularly in ICUs in low- and middle-income countries, where compliance with infection prevention measures tends to be lower, exacerbating cross-transmission [15].

Third, antimicrobial selective pressure enhances the survival and proliferation of resistant strains, particularly in critically ill patients with compromised immune function. These patients frequently develop microbiome disturbance or dysbiosis as a result of antibiotic exposure and preexisting conditions, which reduces the colonization resistance of commensal flora and facilitates the proliferation of resistant opportunistic pathogens [16]. This dysbiotic state further promotes HGT, biofilm development, and quorum sensing, collectively amplifying the dissemination of resistance genes within the patient and across the ICU environment.

The interaction of mobile genetic elements that facilitate genetic exchange, ICU-specific factors such as antibiotic misuse and transmission dynamics, and host-related susceptibilities such as immune dysfunction and microbiome alteration creates a conducive environment for the emergence, amplification, and spread of AMR in critical care settings. Addressing AMR in ICUs requires comprehensive approaches, including stringent AS to optimize antibiotic use, robust IPC to prevent cross-transmission, and measures to maintain or restore microbiome equilibrium and host defenses [17,18]. Table 2 summarizes the common ICU pathogens associated with AMR, along with their major resistance phenotypes and underlying resistance mechanisms [19-21].

**Table 2:** Common ICU pathogens causing antimicrobial resistance, their major resistance phenotypes, and underlying resistance mechanisms.

Pathogen	Gram Stain	Resistance Phenotype	Key Resistance Mechanisms
*Klebsiella pneumoniae* / *Escherichia coli* (ESBL-producing) [19-21]	Gram-negative	ESBL-producing Enterobacterales (ESBL-E)	Enzymatic hydrolysis of extended-spectrum cephalosporins, commonly by CTX-M ESBLs; often associated with additional resistance mechanisms such as porin loss and efflux pump upregulation
*Enterobacter cloacae* complex, *Citrobacter freundii* [19-21]	Gram-negative	AmpC β-lactamase-producing Enterobacterales (AmpC-E)	Chromosomal or plasmid-mediated AmpC β-lactamases hydrolyzing penicillins and cephalosporins; not reliably inhibited by clavulanate
*Klebsiella pneumoniae* (KPC-producing CRE) [19-21]	Gram-negative	Carbapenem-resistant Enterobacterales (CRE), KPC type	Class A serine carbapenemase hydrolyzing carbapenems; compounded by porin loss, such as OmpK35/36 loss, reducing carbapenem entry
*K. pneumoniae* / *E. coli *(NDM-producing CRE) [19-21]	Gram-negative	CRE, metallo-β-lactamase (MBL) type	Metallo-β-lactamase hydrolyzing most β-lactams, including carbapenems, but not aztreonam; not inhibited by avibactam; often associated with efflux and porin loss
*K. pneumoniae* (OXA-48-producing CRE) [19-21]	Gram-negative	CRE, OXA-type carbapenemase	Class D serine carbapenemase with clinically significant carbapenem hydrolysis; activity may be enhanced by ESBL production and porin loss
*Acinetobacter baumannii *(CRAB) [19-21]	Gram-negative	Carbapenem-resistant *A. baumannii *(CRAB)	OXA-type carbapenemases, including OXA-23, OXA-24/40, and OXA-58; AdeABC/AdeIJK efflux pumps; outer membrane protein loss, such as CarO; aminoglycoside-modifying enzymes; biofilm formation
*Pseudomonas aeruginosa* (DTR) [19-21]	Gram-negative	Difficult-to-treat resistance *P. aeruginosa* (DTR-PA)	MexAB-OprM/MexXY-OprM efflux systems; OprD porin loss; acquired β-lactamases, such as VIM and NDM; target mutations, such as GyrA/ParC
*Staphylococcus aureus* (MRSA) [19-21]	Gram-positive	Methicillin-resistant *S. aureus* (MRSA)	Altered penicillin-binding protein PBP2a with low β-lactam affinity; biofilm formation contributes to persistence
*Enterococcus faecium* / *E. faecalis* (VRE) [19-21]	Gram-positive	Vancomycin-resistant Enterococcus (VRE)	D-Ala-D-Ala replaced by D-Ala-D-Lac via *vanA*/*vanB* genes, reducing vancomycin-binding affinity

Pharmacological management of MDR infections

MDR infections pose a major healthcare concern, particularly in ICU settings. Early management is essential and helps reduce associated mortality [22]. Appropriate empiric therapy is required for better outcomes, depending on several factors, such as disease severity, source of infection, immune status, and penicillin allergy. It is always best to consider the patient’s previous antibiotic exposure and antimicrobial susceptibility testing data before selecting the correct therapy [19].

AS involves selecting, administering, de-escalating, and maintaining antimicrobial therapy in a way that maximizes clinical outcomes while limiting adverse patient outcomes and the emergence of resistance [23]. Two main drug regimens are commonly used in the management of MDR infections: monotherapy, which involves the use of a single antimicrobial agent and is generally preferred in clinically stable patients, and combination therapy, which uses two or more antimicrobial agents to improve antimicrobial coverage, enhance therapeutic efficacy, and potentially achieve better clinical outcomes, particularly in critically ill patients.

Therapeutic drug monitoring (TDM) refers to a clinical approach used to measure therapeutic drug levels in blood or plasma at intervals to optimize therapy and reduce the risk of toxicity. TDM makes it possible to evaluate a drug’s effectiveness and safety in a range of clinical contexts by integrating knowledge of pharmaceutics, pharmacokinetics (PK), and pharmacodynamics (PD). PK and PD are two basic elements required for any drug to achieve effectiveness in a patient’s body. In ICU patients, a tailored antimicrobial regimen is preferred because of illness severity and changes in patient physiology.

As mentioned, critically ill patients undergo a number of alterations that frequently result in noticeably varied antibiotic exposures, which affect toxicity and effectiveness. Additionally, the bacterial inoculum, which is typically significantly higher in critically ill patients, may affect the kinetics of bacterial death and may be a factor in treatment failure. Individualization of dosage based on the PK of antibiotics and the patient’s unique clinical features is essential to obtain optimal therapeutic exposure based on bacterial susceptibility, potentially improve bacterial killing, and limit toxicity. Promoting high peak concentrations for concentration-dependent antibiotics by concentrating the daily dose at a single time point or, on the other hand, promoting longer exposure for time-dependent antibiotics, such as penicillins, with prolonged or continuous infusions has been suggested to maximize therapeutic success [24]. With the increasing prevalence of MDR organisms, the right antibiotics must be chosen based on pathogen-specific resistance patterns to maximize outcomes and prevent future resistance.

MRSA

Vancomycin: In adults with normal renal function, IV vancomycin 15-20 mg/kg/dose based on actual body weight every 8-12 hours is recommended, not exceeding 2 g per dose. A loading dose of 25-30 mg/kg based on actual body weight may be considered in critically ill patients with suspected MRSA infection, such as those with sepsis, meningitis, PN, or infective endocarditis. Extending the infusion period to two hours and using an antihistamine before administering the loading dose may be considered to reduce the risk of vancomycin infusion reaction.

For individuals with severe infections, those who are morbidly obese, have renal impairment, including those undergoing dialysis, or have varying volumes of distribution, vancomycin monitoring is advised. Vancomycin trough concentrations of 15-20 µg/mL are advised for serious infections such as bacteremia, infective endocarditis, osteomyelitis, meningitis, PN, and severe SSTI, such as necrotizing fasciitis, caused by MRSA. There is insufficient information to recommend a single vancomycin dosage for children. Children with serious or invasive infections should receive IV vancomycin at a dose of 15 mg/kg every six hours. Regimens comprising continuous infusion of vancomycin are not advocated [25].

Linezolid: Bacteremia and other disorders caused by MRSA, such as PN, osteomyelitis, skin and soft tissue infections, and UTIs, can be effectively treated with linezolid. Even in individuals with complex infections, oral administration is possible due to bioavailability approaching 100% [26].

Daptomycin: The use of daptomycin to treat enterococcal infections, particularly those caused by vancomycin-resistant strains, has grown. There is growing evidence that the EMA-approved daptomycin dosage regimens (4-6 mg/kg/day), which were first developed to treat *Staphylococcus aureus*-associated infections, are inadequate for treating enterococcal BSI [27].

Ceftaroline: A broad-spectrum cephalosporin, ceftaroline fosamil exhibits in vitro activity against a variety of prevalent community-acquired pneumonia (CAP) pathogens, such as MSSA, MRSA, Streptococcus pneumoniae, and non-β-lactamase-producing Enterobacterales. It has a well-established clinical profile in patients with complex skin and soft tissue infections (cSSTIs) and CAP in both adults and children [28].

Vancomycin-Resistant Enterococci (VRE)

Despite being bacteriostatic, linezolid is frequently used because of its oral formulation and consistent efficacy. In severe infections such as bacteremia and endocarditis, daptomycin, a bactericidal drug, is often recommended, frequently at higher doses. Infections that are difficult or persistent may be treated with combination therapy.

ESBL-Producing Organisms

Enzymes called ESBLs render the majority of cephalosporins, aztreonam, and penicillins inactive. In general, ESBL-E often remain susceptible to carbapenems. Non-β-lactam drugs, such as gentamicin, trimethoprim-sulfamethoxazole (TMP-SMX), and ciprofloxacin, are not rendered inactive by ESBLs. Nevertheless, organisms with ESBL genes frequently have extra genes or gene changes that increase their resistance to a variety of antibiotics.

When treating ESBL-E infections outside the urinary tract, a carbapenem is advised [19]. The recommended medications are ertapenem, imipenem-cilastatin, or meropenem; ertapenem provides a more practical choice for patients who must continue carbapenem therapy in an outpatient setting when oral treatment options are unavailable.

Carbapenem-Resistant Enterobacterales (CRE)

CRE pose a significant global public health concern because of limited therapeutic options and high associated mortality, particularly in critically ill individuals. Resistance is most commonly mediated by carbapenemase enzymes or by a combination of ESBL production with porin loss and efflux pump overexpression. Carbapenemase-producing CRE are particularly concerning because of their rapid transmissibility via plasmids [19]. Ceftazidime-avibactam is active against CRE that produce carbapenemase enzymes such as KPC and OXA-48 but not against those that produce metallo-β-lactamases, with studies showing it as a better alternative compared with colistin-based therapy [29]. Meropenem-vaborbactam is effective against KPC-producing CRE, with improved clinical cure rates, reduced mortality, and lower nephrotoxicity. Imipenem-relebactam is also effective against KPC-producing CRE, as it restores carbapenem activity by inhibiting selected β-lactamases [30]. Cefiderocol is active against KPC-, OXA-48-, and metallo-β-lactamase-producing CRE, with favorable efficacy [31].

MDR Pseudomonas aeruginosa

MDR *Pseudomonas aeruginosa* is common in ICU infections, such as ventilator-associated PN, UTI, and BSI, and has high intrinsic and acquired resistance. Cefiderocol has shown clinical utility in selected patients with MDR *Pseudomonas aeruginosa*, although comparative advantages over ceftolozane-tazobactam or ceftazidime-avibactam depend on the susceptibility pattern and clinical context [32].

Infection prevention and control

Several essential elements are involved in IPC strategies in an ICU.

Engaging the Infection Control Team and Key Facilitators or Stakeholders

The execution of IPC programs depends on interdisciplinary collaboration. To oversee the multidisciplinary work group and implement behavioral change, a program leader with excellent interpersonal and organizational abilities must be chosen. Establishing a network of internal advocates and stakeholders who can track advancement in real time and sustain high levels of attention through frequent feedback is also crucial in the ICU [33].

HAIs, Alert Organisms, and Ongoing HAI Surveillance

BSI and PN must be included in HAI surveillance of ICU-acquired infections. Furthermore, it is crucial to have a real-time automated detection system for tracking MDROs and to frequently update cumulative antibiograms that include all ICU microbiological isolates [34,35].

Hand Hygiene

The foundation of all IPC initiatives is improved performance feedback to increase hand hygiene compliance in the ICU [36].

Active Screening Techniques

Active surveillance cultures (ASCs) can be very helpful in predicting invasive infections and directing focused, vertical prevention tactics in environments where MDR bacteria are highly prevalent. ASCs have been used either broadly or specifically for certain patient populations. Appropriate antimicrobial therapy has also been guided by ASCs [37,38].

Contact Precautions

There is debate on the advantages of universal gloves and gowns, particularly in ICUs. Based on local epidemiology, patient vulnerability, effect sizes, adherence to recommended precautions, resources, and implementation costs, each hospital should evaluate the intervention [39].

Decontamination Techniques

Daily bathing of ICU patients with washcloths soaked with 2% chlorhexidine may lower the incidence of healthcare-associated infections (HAIs) caused by both Gram-positive and Gram-negative MDROs. Targeted decolonization techniques, on the other hand, should be customized to each patient’s unique risk profile, including microbiota composition, clinical severity (ECMO, bowel hypoperfusion), and comorbidities (immunocompromised or transplanted). A customized approach is highly advised, as discussed in the following paragraph.

Environmental Disinfection

No-touch decontamination (NTD) technologies should not replace improved cleaning and disinfection with standard products and external monitoring. However, NTD might be helpful in terminal or discharge cleaning to reduce staff workload and minimize exposure to infection reservoirs [40].

Care Bundles

To avoid HAIs, infection control bundles in the ICU are organized into several sets of evidence-based procedures. These bundles may be customized for high-priority MDROs or target certain ICU-related HAIs, such as ventilator-associated PN, catheter-associated UTIs, and central line-associated BSI. Each bundle’s efficacy depends on how well it is implemented and how frequently frontline staff receive feedback [41].

Teaching and Training

Healthcare simulation (HCS) is increasingly being used for medical teaching, particularly in ICUs. HCS is especially well suited to safely learning and practicing both technical and team skills required for critical care practice, since patients in the ICU require invasive procedures and effective interprofessional team performance in a high-stakes setting with potential consequences [42].

AS

IPC and AS necessitate coordinated, interdependent action across several overlapping healthcare settings and disciplines. To accomplish the greater goal of protecting patients from infection and ensuring that effective antibiotic therapy is available for future generations, IPC and AS program directors will need to take deliberate strategic relationship-building efforts [43]. Table 3 shows a risk stratification framework for prioritization of MDRO prevention and control in ICU settings.

**Table 3:** Risk stratification framework for prioritization of multidrug-resistant organism prevention and control in ICU settings. IPC: Infection prevention and control; PDR: Pan-drug-resistant; XDR: Extensively drug-resistant; MDR: Multidrug-resistant.

Characteristic	High Priority	Medium Priority	Low Priority
Facility factors [43]	Poor hand-hygiene adherence (<50%); open-plan intensive care setting; patient overcrowding	Moderate hand-hygiene adherence (>50%); partially resourced IPC protocols; restricted availability of single rooms	Strong hand-hygiene adherence (>70%); formally audited IPC program; single-room capacity available
Pathogen factors [43]	Rapidly spreading agent; survives well in the environment; high case fatality; epidemic spread pattern; pan-drug-resistant (PDR) organism	Self-contained outbreak pattern; intermediate mortality burden; intermediate transmission efficiency; environmental persistence >1 week; extensively drug-resistant (XDR) organism	Limited spread potential; low lethality; short environmental survival (<1 day); endemic baseline presence; multidrug-resistant (MDR) organism
Patient factors [43]	Active symptomatic infection; compromised immune function; elevated intrinsic healthcare-related risk; significant underlying conditions	Complex medical intervention history; colonization at multiple body sites; prior hospital admission; extended ICU stay	Clinically well, no active illness Asymptomatic colonisation only Minimal extrinsic healthcare exposure Brief ICU stay (< few weeks)

Challenges, innovations and future perspectives

The management of ICU infections is made more difficult by the continuous emergence of novel MDROs, which compromises treatment efficacy and worsens patient outcomes. ICUs serve as amplification sites for AMR because of the high concentration of vulnerable patients and complex care practices [44]. Despite advances in antimicrobial therapy, multidrug resistance remains a major global health challenge, particularly in ICU settings where antibiotic pressure is high. It is projected that by 2050, there could be approximately 10 million deaths annually attributed to antibiotic-resistant infections, making AMR one of the leading causes of mortality worldwide [45].

AMR remains a major challenge in critical care due to the rapid dissemination of MDR pathogens and the scarcity of novel therapeutic options. The intensive use of antibiotics, coupled with vulnerable patient populations and invasive interventions, creates an environment that promotes the selection and transmission of resistant microorganisms [44]. These challenges are further exacerbated in low- and middle-income countries, where limited diagnostic capacity, inadequate infection control measures, and restricted access to newer antimicrobials contribute to a higher MDR burden [45]. Unregulated antibiotic sales, antibiotic overuse and misuse, inadequate healthcare infrastructure, inadequate infection control, and environmental pollution are other causes of AMR in low- and middle-income countries. The issue is made worse by socioeconomic factors, including self-medication due to restricted access to healthcare [46,47].

Emerging technologies such as artificial intelligence are increasingly being explored to guide antimicrobial selection, predict resistance patterns, and optimize treatment decisions in real time [45]. Personalized dosing strategies using pharmacokinetic and PD modeling are particularly important in critically ill patients, in whom altered drug distribution and clearance can significantly affect therapeutic outcomes. Combination therapies should be considered in selected patients with septic shock and high-risk ICU patients [48]. Advanced neural networks and predictive analytics may be able to detect high-probability infections or positive cultures earlier, enabling more rapid, focused treatment [49].

Innovative strategies such as antimicrobial peptides, nanotechnology-driven drug delivery systems, and microbiome-targeted therapies present promising opportunities for addressing resistance. These approaches target bacterial membranes and are highly sought after because they are effective against slow-growing bacteria and biofilms, have high stability in serum and good tissue penetration, can enhance the activity of many antibiotics, and have a low potential for pathogen resistance development [50]. Preventive measures, especially vaccinations, are essential in decreasing infection rates and minimizing antibiotic consumption, thereby limiting resistance development. Governments and businesses must concentrate on creating innovative medications and vaccines to combat resistant infections [51].

Ultimately, economic factors and cost-effectiveness are pivotal elements affecting the accessibility and implementation of these advanced therapies, especially in low- and middle-income countries, where global coordination is of paramount importance [47]. Surveillance systems and AS programs are needed to ensure that ongoing efforts can match the evolutionary capacity of these microbes and tackle local emergence and global spread of AMR. Continued investment in research, along with international collaborations in data collection, advancing AI models, and genomic studies, will be critical to counter the evolving threat of MDR pathogens [50].

Clinical recommendations on managing MDR infections in ICUs

The growing problem of MDR infections in ICUs calls for the implementation of evidence-based approaches that combine pharmacological and non-pharmacological treatments. A multidisciplinary approach is essential to improve patient outcomes, reduce transmission, and lessen the overall burden of MDR infections because of the high incidence of resistant bacteria, the complexity of critically ill patients, and the need for prompt and effective treatment.

Evidence-Based Algorithms for Managing MDR Infections in ICUs

Evidence-based, patient-tailored algorithms that incorporate rapid diagnosis, risk assessment, and optimal antibiotic therapy are necessary for the effective management of MDR infections in the ICU. Current guidelines emphasize the need for timely, appropriate antibiotic therapy informed by pathogen susceptibility profiles, individual patient risk factors, and local epidemiology. Optimal management of AMR in the ICU also requires a multifaceted approach that integrates timely empiric antibiotic administration, knowledge of local resistance patterns, rapid diagnostic testing, AS, and early de-escalation strategies. Individual patient risk factors, prior antimicrobial exposure, colonization status, and local epidemiological data should guide antibiotic selection to maximize treatment success while minimizing the emergence of resistance [52,53].

New β-lactam/β-lactamase inhibitor combinations, such as ceftolozane-tazobactam and ceftazidime-avibactam, have been effective against MDR *Pseudomonas aeruginosa* and other resistant Gram-negative infections when susceptible, leading to changes in treatment algorithms [2]. In critically ill patients with suspected MDR infections, combination therapy may be considered empirically until susceptibility results are known; once targeted therapy is established, de-escalation to monotherapy may occur [54].

Current methods incorporate antimicrobial susceptibility testing and rapid diagnostic technologies to shorten the duration of ineffective empiric treatment and lessen selection pressure [55]. The use of PK/PD optimization and TDM improves antibiotic exposure adequacy, particularly in critically ill patients with altered drug clearance [55]. Several randomized controlled trials have demonstrated enhanced microbiological eradication and clinical cure rates using optimized dosing regimens, such as continuous infusions and TDM-guided therapy, especially in patients with sepsis and septic shock [56].

Integration of Pharmacological and Non-Pharmacological Strategies

Multimodal approaches that integrate pharmacological therapies with non-pharmacological measures provide the optimal means to manage MDR infections in the ICU. Pharmacologically, in addition to traditional and newer antibiotics, adjuvant therapies such as phage therapy are developing as targeted alternatives, providing potential for biofilm destruction and synergy with antibiotics to restore bacterial susceptibility [57]. However, phage therapy remains experimental and requires further clinical validation and regulatory approval in critically ill populations.

Non-pharmacological strategies, including stringent IPC, AS programs (ASPs), and environmental cleaning, are fundamental in limiting the transmission of MDR microorganisms. Comprehensive preventive bundles in the ICU that encompass hand hygiene, contact precautions, environmental sanitation, and reduced use of invasive devices can reduce the spread of MDROs [58]. Evidence-based policy guidelines advocate for tiered targeted interventions integrated into ICU workflows to disrupt transmission chains and protect patient outcomes [58].

In critically ill patients and ICU environments, stewardship programs that incorporate rapid diagnostics alongside structured protocols for dose optimization and early de-escalation have demonstrated, through randomized studies and systematic reviews, reduced AMR rates and adverse events while preserving therapeutic efficacy [59,60]. These programs frequently employ daily evaluations of antibiotic therapy, informed by culture results and PK/PD factors, to customize the duration and spectrum of treatment.

In summary, substantial evidence from recent randomized controlled trials and guideline-based research supports AS strategies that integrate antibiotic de-escalation, dosage optimization, and suitable treatment durations as essential elements to reduce the development of resistance. These tactics, enhanced by rapid diagnostics and incorporated into clinical workflows, constitute the foundation of effective stewardship interventions across various infectious diseases and healthcare environments [60-62].

Emphasis on Multidisciplinary Teamwork

Multidisciplinary teamwork is crucial for the effective management of MDR infections in ICU environments. Infectious disease specialists, intensivists, clinical microbiologists, pharmacists, infection control practitioners, and nursing personnel must collaborate to ensure prompt pathogen identification, antibiotic stewardship, and compliance with IPC guidelines [58]. Regular multidisciplinary meetings enable the assessment of antimicrobial susceptibilities, the execution of treatment protocols, and the appraisal of infection control efficacy. Pharmacists play a critical role in PK/PD optimization and antimicrobial dosing adjustments based on TDM, especially in the variable physiology of critically ill patients [54]. Nursing staff are frontline agents in preventing device-associated infections and maintaining IPC adherence, critically influencing infection incidence and transmission dynamics [58].

Key Practice Points and Take-Home Messages

Early recognition and risk stratification are essential in the management of MDR infections, as prompt identification of patients at risk helps guide appropriate empiric antimicrobial therapy and facilitates timely implementation of isolation measures. Tailored antimicrobial therapy should be initiated based on local epidemiological patterns and common MDR pathogens, with early de-escalation according to susceptibility results to reduce toxicity and limit further resistance selection [53,54]. The use of novel antimicrobial agents, including newer β-lactam/β-lactamase inhibitor combinations, has shown benefit in treating resistant Gram-negative infections, while aminoglycosides may remain an effective therapeutic option, particularly in UTIs caused by MDR organisms [55]. Adjunctive therapies such as bacteriophage therapy may also be considered cautiously in selected cases, although further clinical evidence is required to establish their routine use [56]. AS programs play a critical role in optimizing antibiotic therapy through strategies such as TDM and PK/PD principles. In addition, comprehensive infection control measures, including strict hand hygiene, environmental cleaning, and contact precautions, are necessary to prevent transmission of MDR pathogens in ICU settings [58]. A multidisciplinary approach involving intensivists, infectious disease specialists, microbiologists, pharmacists, and nursing staff is essential to ensure comprehensive and effective management of MDR infections [55].

Policy Implications and Research Priorities

From a policy standpoint, to effectively manage MDR organisms in ICU settings, healthcare institutions must create and support clear policies that combine evidence-based AS, infection control tactics, and rapid diagnostic tools. Policymaking should encourage the allocation of funds for education, ongoing monitoring, and the incorporation of innovative treatments within well-regulated frameworks [58].

Research Priorities Highlighted by Systematic Analyses and Guidelines

Further research is needed to optimize antibiotic dosing strategies, treatment duration, and the use of combination versus monotherapy regimens for MDR infections in ICUs through well-designed randomized controlled trials. The clinical efficacy and safety of emerging therapeutic approaches, including bacteriophage therapy and newer antimicrobial agents, should also be evaluated in critically ill patients to determine their role in routine clinical practice. In addition, the development of rapid point-of-care diagnostic tools is essential to enable timely pathogen identification and resistance profiling, thereby supporting personalized antimicrobial therapy and improving clinical outcomes. Future studies should also focus on implementation science strategies aimed at improving adherence to AS protocols and strengthening infection prevention practices in ICU settings. Furthermore, evaluating the cost-effectiveness of intervention bundles and assessing the economic burden of MDRO infections across different intensive care settings, particularly in resource-limited regions, remains crucial for informing policy decisions and optimizing healthcare resource allocation [55-58,63].

## Conclusions

MDR infections in intensive care units remain a major global health challenge, contributing significantly to morbidity, mortality, prolonged hospital stays, and increased healthcare costs. The complex ICU environment, frequent use of invasive devices, and high antimicrobial exposure promote the emergence and spread of resistant pathogens, making early recognition and timely management essential. Effective control requires an evidence-based multidisciplinary approach that integrates rapid diagnosis, appropriate antimicrobial therapy with timely de-escalation, PK and PD optimization, AS, and strict IPC measures. Emerging advances, such as rapid diagnostics, novel antimicrobial agents, and innovative therapies, offer promising future strategies; however, continued research, stronger stewardship programs, and coordinated multidisciplinary efforts are essential to improve patient outcomes and preserve antimicrobial effectiveness in critical care settings.
